# Complete Growth Inhibition of *Pseudomonas aeruginosa* by Organo-Selenium-Incorporated Urinary Catheter Material

**DOI:** 10.3390/antibiotics13080736

**Published:** 2024-08-06

**Authors:** Phat L. Tran, Caroline L. Presson, Md Nayeem Hasan Kashem, Wei Li, Ted W. Reid, Werner T. W. de Riese

**Affiliations:** 1 Department of Ophthalmology and Visual Sciences, Texas Tech University Health Sciences Center, Lubbock, TX 79430, USA; phat.tran@ttuhsc.edu; 2 Department of Urology, School of Medicine, Texas Tech University Health Sciences Center, Lubbock, TX 79430, USA; caroline.presson@ttuhsc.edu (C.L.P.); werner.deriese@ttuhsc.edu (W.T.W.d.R.); 3 Department of Chemical Engineering, Texas Tech University, Lubbock, TX 79409, USA; md-nayeem-hasan.kashem@ttu.edu (M.N.H.K.); wei.li@ttu.edu (W.L.)

**Keywords:** catheter-associated urinary tract infection, selenium, *Pseudomonas aeruginosa*, in vitro growth inhibition

## Abstract

To further investigate the inhibition of *Pseudomonas aeruginosa*’s in vitro growth and biofilm formation by an organo-selenium-incorporated polyurethane (PU) catheter material. *P. aeruginosa*, *Staphylococcus aureus*, and *Candida albicans* were incubated in vitro with organo-selenium and control polyurethane catheter materials in the presence of glutathione. Growth was evaluated by a colony-forming-unit (CFU) count and visualized with confocal laser scanning microscopy. Two different PU catheter materials were used. Using tin-catalyzed PU catheter material, complete inhibition of *S. aureus* was seen at 1% selenium (Se), whereas no inhibition was seen for *P. aeruginosa* at up to 3.0% Se. Whereas, using a thermoplastic PU catheter material, 1.5% Se and 2% Se organo-selenium caused several logs of growth inhibition of *P. aeruginosa*, and 2.5% selenium, incorporation showed complete inhibition (8 logs). Samples with lower than 1.5% selenium did not show adequate growth inhibition for *P. aeruginosa*. Similar in vitro growth inhibition was achieved against a multidrug-resistant *C. albicans* strain. It was concluded that optimal inhibition of *P. aeruginosa* in vitro growth and biofilm formation occurs with 2.5% selenium incorporated as organo-selenium in a thermoplastic PU catheter material. These results suggest that reduced incidence of CAUTIs (catheter associated urinary tract infections) with *P. aeruginosa* and other bacteria and fungi can be achieved by using organo-selenium-incorporated catheters.

## 1. Introduction

Urinary catheters are frequently used in hospitalized patients and cause one of the most prevalent infections, called catheter-associated urinary tract infections (CAUTIs) [1]. Urinary tract infections are the fourth most common nosocomial infection in healthcare settings [2]. The most common organisms responsible for CAUTIs are *Escherichia coli*, *Klebsiella pneumoniae*, *Staphylococcus aureus*, *Candida albicans*, and *Pseudomonas aeruginosa* (in more than 90–95%) [2,3]. Multiple attempts with antiseptic and antibiotic-coated catheters have been made to lower the incidence of CAUTIs by controlling colonization and biofilm formation [4]; however, their use has not become the standard of care [5].

**Figure 1:**
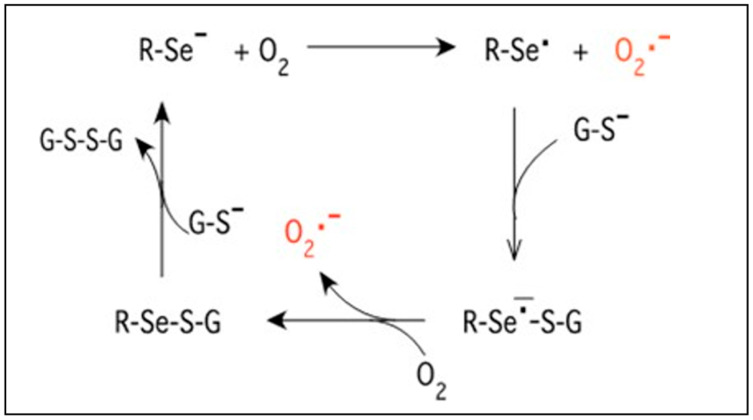
Equation of the selenide anion reduction pathway for generating superoxide.

An interesting new approach of using organo-selenium-incorporated catheters has demonstrated excellent inhibition of growth and biofilm formation for bacteria [6]. Selenium has the unique ability to catalyze the formation of superoxide radicals as outlined in Figure 1. Selenium takes an electron from sulfur-containing molecules such as glutathione, which is universally available in all biological tissues, and gives it to oxygen, resulting in the formation of a superoxide radical. This radical causes oxidative stress for bacterial cells, with secondary cell death [7]. These radicals have a short half-life due to fast dismutation and, thus, have no adverse effect on the surrounding human tissue. The bacterial growth and biofilm inhibition and the lack of in vivo toxicity are well documented in studies on organo-selenium-coated contact lenses, tooth sealant, and hemodialysis catheters [8,9,10]. A recent study using 1% selenium-incorporated catheter tubing has shown excellent inhibition of growth and biofilm formation for *E. coli*, *K. pneumonia*, *Haemophilus influenzae*, and *S. aureus*, but it failed for *P. aeruginosa* [6]. Thus, this study focuses on a dose–response study to provide adequate in vitro inhibition of *P. aeruginosa* as well as *C. albicans*. 

## 2. Results

### 2.1. Staphylococcus aureus Growth on Thermoplastic and Tin-Catalyzed Polyurethane

**Figure 2:**
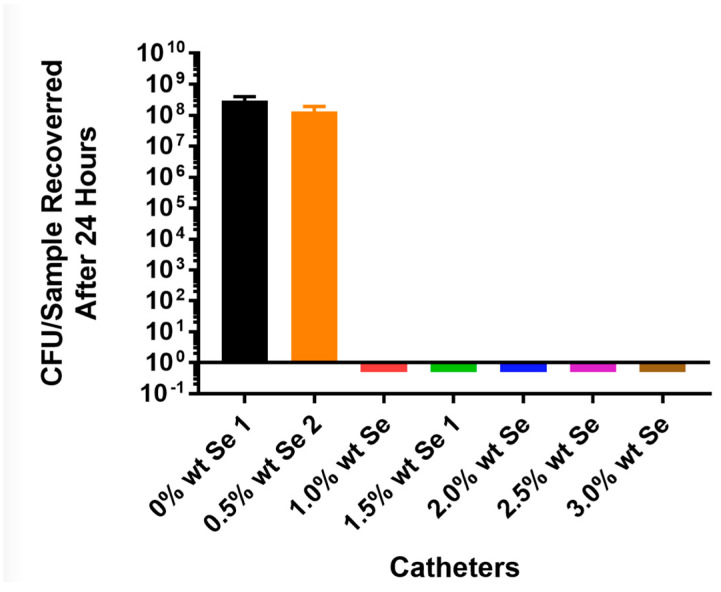
Growth of *Staphylococcus aureus* AH133 green fluorescent protein (GFP) on catheter tubing without and with different concentrations of selenium incorporated using a tin catalyst.

**Figure 3:**
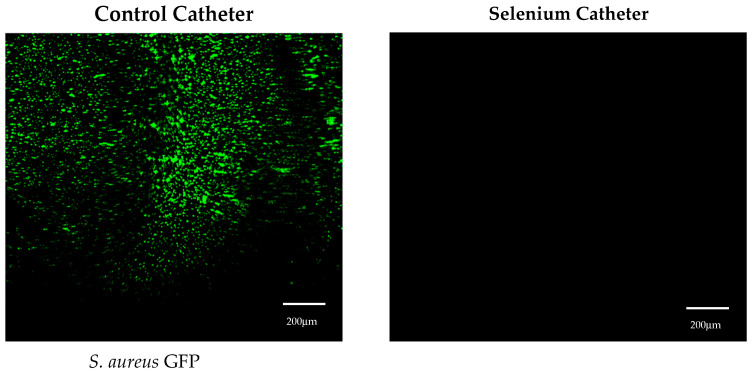
Visualization of surfaces by confocal laser scanning microscopy (CLSM) in control and 1% selenium catheter tubing following 24 h incubation with *Staphylococcus aureus* strain GFP AH133.

For *Staphylococcus aureus*, the bacteria grew over 7 logs in 24 h on the control tubing, while *S. aureus* did not grow on the tin-catalyzed selenium polyurethane at a 1% selenium concentration (Figure 2). This was also demonstrated by the confocal laser scanning microscopy (CLSM) visualization of the thermoplastic polyurethane surfaces in control and organo-selenium-coated catheters following a 24 h bacterial incubation with *S. aureus* (Figure 3).

### 2.2. P. aeruginosa Growth on Polyurethane Using a Tin Catalyst

**Figure 4:**
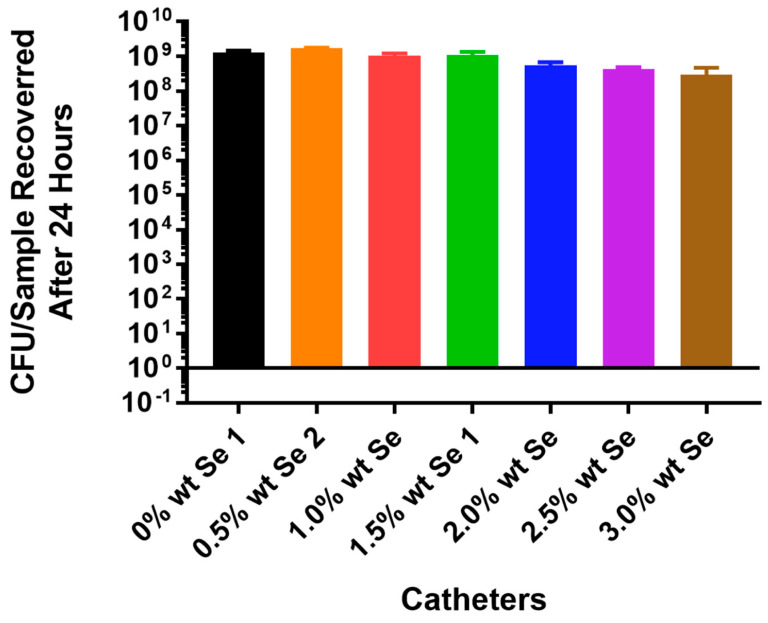
Growth of *Pseudomonas aeruginosa* PA01 GFP on catheter tubing without and with different concentrations of selenium incorporated using a tin catalyst.

The in vitro results for *P. aeruginosa* obtained with the PU samples using tin as a metal catalyst showed no inhibition of cell growth at concentrations of selenium up to 3% of the polymer (Figure 4). These results were very different compared to the results obtained with *Staphylococcus aureus* in which total inhibition was seen with only 1% selenium in the polymer.

### 2.3. P. aeruginosa Growth on Thermoplastic Polyurethane

**Figure 5:**
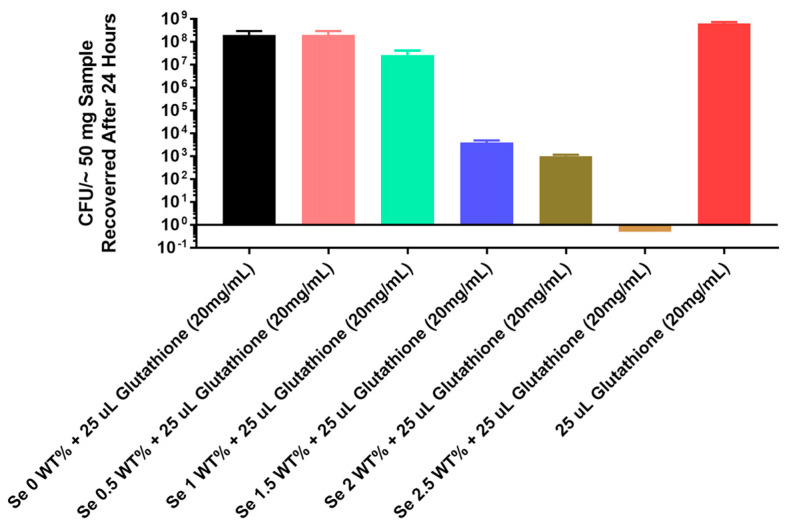
Growth of *Pseudomonas aeruginosa* PA01 GFP on catheter tubing without and with different concentrations of selenium incorporated using a thermoplastic monomer.

The results for the thermoplastic PU are seen in Figure 5: the 1.5% by weight selenium showed approximately 4 logs of inhibition and the 2% selenium showed 5 logs of inhibition; the 2.5% selenium showed 8 logs of inhibition (total growth inhibition). 

### 2.4. Candida albicans Growth on Thermoplastic Polyurethane 

**Figure 6:**
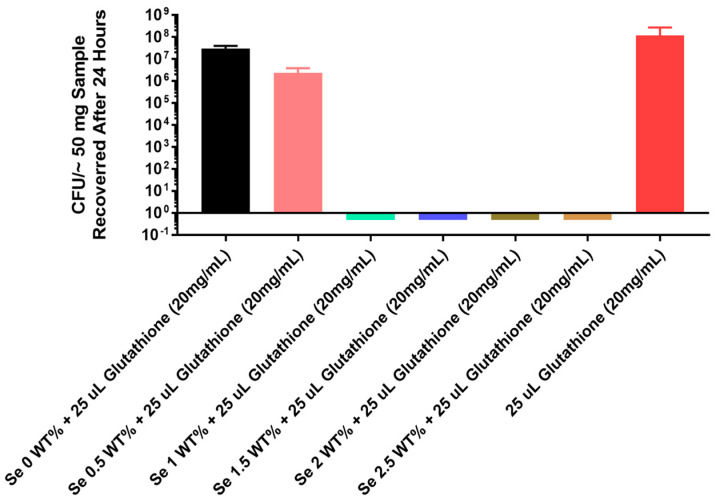
Growth of the multidrug-resistant strain *Candida albicans* ATCC 10231 on catheter tubing without and with different concentrations of selenium incorporated using a thermoplastic monomer.

The thermoplastic polyurethane catheter also showed complete inhibition (8 logs) of in vitro growth of the multidrug-resistant fungal strain of *C. albicans* even at a 1% selenium concentration as seen in Figure 6. 

## 3. Discussion

As is well known and documented in healthcare, urinary tract infections (UTIs) are one of the most common nosocomial infections. Up to 80% of UTIs are due to indwelling catheters, also called catheter-associated UTIs (CAUTIs), as they provide an attachment surface for microbial adhesion and ascending migration [11]. Previous studies have attempted to use different antiseptic and antimicrobial coatings to inhibit bacterial growth and biofilm formation and thus prevent CAUTIs [3]. Coating technologies covering only the surface of the catheter material, as implemented with antibiotics, have brought up concerns such as substance instability and leaching over time [12]. Therefore, complete incorporation by polymerization of the antimicrobial substance within the catheter material (as implemented in this study with an organo-selenium-polymerized catheter material) is considered superior to more superficial, less stable modifications such as coating techniques. Furthermore, the previous studies on selenium-incorporated catheters [6] have demonstrated consistent antimicrobial efficacy for 12 weeks under in vivo conditions, which is reassuring as indwelling catheters are currently replaced every 4 weeks.

Nosocomial UTIs often have unusual antibiotic-resistance patterns; therefore, cultures are recommended for appropriate antibiotic selection in covering the pathogen. Furthermore, best practice limits the duration of antibiotic use in order to lower the chances of developing drug-resistant bacteria under treatment [1]. Despite these measures drug-resistant bacteria are causing increasing clinical issues in CAUTIs. In this context, catheters with direct antimicrobial properties, such as organo-selenium-incorporated catheters, may be very advantageous in preventing bacterial colonization and biofilm formation.

Human clinical trials have demonstrated that organo-selenium coatings on teeth are non-toxic to the surrounding gum tissue after a year [13], and animal trials using selenium-coated contact lenses showed no effect on the cornea after 3 months [8]. This supports the idea of implementing organo-selenium-incorporated catheters in future clinical trials. Previous in vitro studies have also demonstrated that 1% organo-selenium-incorporated catheters provide excellent growth inhibition for *E. coli*, *K. pneumoniae*, *S. aureus*, and *H. influenzae* but have failed for *P. aeruginosa* [6]. The current in vitro study analyzes modified organo-selenium concentrations for inhibiting *P. aeruginosa* growth and biofilm formation.

In attempting to incorporate organo-selenium into PU, we used a tin catalyst. As seen in Figure 2, at concentrations of selenium as low as 1%, this polymer resulted in the complete (8 logs) of inhibition of the attachment of *Staphylococcus aureus* to the polymer. However, even at concentrations as high as 3%, selenium showed no inhibition of *P. aeruginosa* (Figure 4) in the tin-catalyzed polymer. Apparently, tin added to selenium caused some inhibition of the ability of selenium to catalyze the formation of superoxide radicals. Thus, *Pseudomonas aeruginosa*, which requires higher concentrations of superoxide, was not adequately growth inhibited in the presence of the tin-catalyzed polymer. 

Considering these results with the tin catalyst, we tried a thermoplastic method of polymerizing the incorporation of the organo-selenium into PU. The study inhibition data for thermoplastic incorporation in Figure 5 show that complete inhibition (8 logs) was seen with 2.5% selenium. 

From a clinical standpoint, it is also interesting that this thermoplastic catheter material optimally inhibited the in vitro growth of the multidrug-resistant fungal strain of Candida albicans even at a 1% selenium concentration, as seen in Figure 6.

Based on the above results, organo-selenium-incorporated catheters should be studied further in vivo, as well as clinically investigated, to determine their therapeutic potential in preventing CAUTIs. While the presented results are promising, we are aware of relevant limitations. Although the presented results of in vitro growth and biofilm inhibition suggest a reduction in CAUTIs caused by *P. aeruginosa*, future clinical studies in patients with indwelling catheters are still needed to prove this clinical assumption. Additionally, while previous in vivo studies have well demonstrated the non-toxicity of selenium in other, non-urological medical devices, future in vivo studies on organo-selenium-incorporated catheters must confirm this prior to implementing clinical trials in patients.

Other studies with selenium and devices to inhibit biofilms on devices have used different methods to add the selenium. One study used selenium nanoparticles to coat a polycarbonate device [14], one used ZnO/selenium nanoparticles embedded in a chitosan-based anti-bacterial wound dressing [15], one coated sodium selenite on titanium orthopedic implants [16], and two used selenium nanoparticles to coat medical devices [17,18]. The problem with these methods is that, in each case, the selenium was not covalently attached to the device. Thus, with time it will leach off. This is not the case with the current study because the organo-selenium is covalently polymerized into the polymeric material of the catheter.

## 4. Materials and Methods

### 4.1. Selenium-Incorporated Polyurethane

Organo-selenium was polymerized into polyurethane using two different methods. One method used a thermoplastic polyurethane resin, and the second method used tin as a catalyst. In each case, the organo-selenium molecule (this molecule was a diol) was added to the polyurethane resin to produce a mixture that contained a specific percent by weight of selenium. The weight percent of the selenium ranged from 0% to 3%. This was the weight of the selenium atom and not of the selenium-containing molecule. The percentage of selenium in the diol molecule used to coat the pellets was 52%. The coated pellets were processed and polymerized with the organo-selenium into polyurethane, where the organo-selenium formed a co-polymer. The material was then tested for chemical and microbiological properties. The control non-selenium material was produced by the same process as the selenium-containing material. 

### 4.2. Bacterial Strains, Media, and Growth Conditions

The bacterial strains tested were *Staphylococcus aureus* strain GFP AH133 and *Pseudomonas aeruginosa* PAO1 GFP strain MM294, both of which express a green fluorescent protein from the plasmids pCM11 and pMRP9-1, respectively. Additionally, we also tested the multidrug-resistant *Candida albicans* strain ATCC 10231. The tested strains were grown in Luria Bertani (LB) broth at 37 °C with shaking (250 rpm) except for Candida albicans, which was gown in yeast extract peptone dextrose (YDP). The *S. aureus* GFP AH1333 strain was used as a control with excellent in vitro growth inhibition when exposed to the 1% organo-selenium-incorporated catheter material as proven in a recent study [6]. This *Staphylococcus* strain incorporates the pCM11 plasmid, in which the gene for the green fluorescent protein (GFP) is constitutively expressed [9]. The *P. aeruginosa* strain PAO1/pMRP9-1, which was used, also constitutively expresses the green fluorescent protein from plasmid pMRP9-1. All the studied strains were grown in LB broth at 37 °C with shaking (250 rpm) except that for *Candida albicans*, which was grown in YPD. Biofilm formation was examined using trypticase soy broth (TSB) (MP Biomedicals, Solon, OH, USA) as the medium. To maintain pCM11 in *S. aureus* GFP AH1333, both LB and TSB broths were supplemented with erythromycin (1 µg/mL), and to maintain pMRP9-1 in *P. aeruginosa* strain PAO1/pMRP9-1, the media were supplemented with 300 µg/mL carbenicillin.

### 4.3. Colony Forming Unit (CFU) Determination

This was achieved as previously described, with some modifications [9]. Two pieces (1 cm in diameter and 3 mm thick) of the control or selenium-PU (polyurethane) were placed into individual wells of sterile Falcon 24-well polystyrene plates (Becton Dickinson Labware, Bedford, MA, USA). Each well contained 1 mL of TSB (tryptic soy broth) inoculated with approximately 10^3^ to 10^4^ colony forming units (CFUs) of bacteria, either *S. aureus* or *P. aeruginosa*. Three wells containing TSB only were used as negative controls. The plates were covered and incubated at 37 °C for 24 h with slight shaking on a Titer Plate Shaker (Lab-line Instruments; Melrose Park, IL, USA). For *Candida albicans* [strain 3147 (ATCC 10231), 10^3^ to 10^4^ colony forming units (CFUs) in one mL of YPD (yeast extract peptone dextrose) were inoculated into each well and incubated at 37 °C for 48 h. Three wells containing only YPD were used as negative controls. The biofilms were quantified by determining the number of microorganisms (CFUs) per two pieces. To determine the CFUs, the two pieces were carefully removed from each well of the 24-well plates with sterile forceps, rinsed gently several times with distilled H_2_O, and placed into a 15 mL conical tube containing 2 mL PBS. The tubes were vigorously vortexed three times, for 2–3 min each time, to disrupt the biofilm and detach the bacteria from the pieces. Suspended cells were then serially diluted (10-fold) in PBS and 10 µL aliquots of each dilution were spotted on LB agar plates for *S. aureus* and *P. aeruginosa* or YPD agar plates for *Candida albicans*. The plates were incubated at 37 °C for 24 h, and the number of CFUs was counted. The number of CFU per two pieces was determined using the following formula: CFU counted × dilution factor × 200. The experiments were performed in triplicate. This procedure was completed for all reported selenium concentrations.

### 4.4. Biofilm Image Quantification

The biofilms were visualized using confocal laser scanning microscopy (CLSM) [19]. The in vitro biofilms were developed as described above. After 24 h of incubation at 37 °C, the pieces were gently rinsed to remove loosely attached bacteria. Visualization of the biofilms was accomplished by using a Nikon A1+/AIR+ confocal microscope (Nikon Inc., Melville, NY, USA) with images acquired at 2 µm intervals through the biofilms. Two-dimensional images were acquired using the Nis Elements Imaging software v. 4.20 (Nikon Inc., Melville, NY, USA). The experiments were performed in triplicate.

### 4.5. Graphing Analysis

The results of the CFU assays were graphed with Prism^®^ version 4.03 (GraphPad Software, San Diego, CA, USA). All the experiments were performed in triplicate.

## 5. Conclusions

Urinary tract infections associated with indwelling catheters are very common nosocomial infections causing significant morbidity, mortality, and increased healthcare costs. Thus, different antiseptic and antimicrobial catheter coatings have been previously investigated to inhibit bacterial growth and biofilm formation. These antimicrobial catheter coating techniques have proven effective in lowering the incidence of CAUTIs; however, their clinical implementation has been limited by many factors, preventing their wide acceptance in current clinical practice.

Recent studies with organo-selenium-incorporated catheters confirmed excellent inhibition of growth and biofilm formation for *E. coli*, *K. pneumoniae*, *S. aureus*, and *H. influenzae* but failed for *Pseudomonas aeruginosa*. The presented in vitro study results demonstrate that organo-selenium-incorporated catheter tubing added with glutathione inhibits bacterial attachment, growth, and biofilm formation of *P. aeruginosa* and the previously mentioned bacteria, as well as *Candida albicans*. Glutathione is available throughout the body, and it functions as an electron donor for selenium, which is required for its antimicrobial activity. Our study results should encourage other investigators to proceed with in vivo and randomized clinical trials in order to further verify the prophylactic value of organo-selenium-incorporated catheters with the long-term clinical goal of reducing complications associated with CAUTIs. We plan to carry out these in vivo studies.

## Data Availability

Data are contained within the article.
